# Ultrastructural Aspects of Photodynamic Inactivation of Highly Pathogenic Avian H5N8 Influenza Virus

**DOI:** 10.3390/v11100955

**Published:** 2019-10-16

**Authors:** Denis Korneev, Olga Kurskaya, Kirill Sharshov, Justin Eastwood, Marina Strakhovskaya

**Affiliations:** 1 School of Biological Sciences, Monash University, 25 Rainforest Walk, Clayton, VI 3800, Australia; 2 Federal Research Center of Fundamental and Translational Medicine (CFTM), 630117 Novosibirsk, Russia; 3 Department of Biophysics, Faculty of Biology, M.V. Lomonosov Moscow State University, 119991 Moscow, Russia; 4 Federal Research and Clinical Center of Specialized Medical Care and Medical Technologies, FMBA, 115682 Moscow, Russia

**Keywords:** influenza, H5N8, photodynamic inactivation, photosensitizer, transmission electron microscopy

## Abstract

Ultrastructural studies revealing morphological differences between intact and photodynamically inactivated virions can point to inactivation mechanisms and molecular targets. Using influenza as a model system, we show that photodynamic virus inactivation is possible without total virion destruction. Indeed, irradiation with a relatively low concentration of the photosensitizer (octacationic octakis(cholinyl) zinc phthalocyanine) inactivated viral particles (the virus titer was determined in Madin Darby Canine Kidney (MDCK) cells) but did not destroy them. Transmission electron microscopy (TEM) revealed that virion membranes kept structural integrity but lost their surface glycoproteins. Such structures are known as “bald” virions, which were first described as a result of protease treatment. At a higher photosensitizer concentration, the lipid membranes were also destroyed. Therefore, photodynamic inactivation of influenza virus initially results from surface protein removal, followed by complete virion destruction. This study suggests that photodynamic treatment can be used to manufacture “bald” virions for experimental purposes. Photodynamic inactivation is based on the production of reactive oxygen species which attack and destroy biomolecules. Thus, the results of this study can potentially apply to other enveloped viruses and sources of singlet oxygen.

## 1. Introduction

Photodynamic inactivation (PDI) can be used to destroy pathogenic microorganisms including bacteria, viruses, fungi, and protozoa [1,2,3] which has practical applications for virus-associated lesions treatment [4] and the disinfection of water [5] or blood products [6]. The PDI technique works by exposing microorganisms to a photosensitizer which, when irradiated with the spectral region corresponding to the photosensitizer absorption bands, converts molecular oxygen into toxic reactive oxygen species (ROS). Inactivation then occurs as a result of the destructive action of ROS produced by the photosensitizer in a photoexcited state.

Enveloped viruses (e.g., Influenza viruses, Hunan Immunodeficiency Virus, and Herpes Simplex Virus) can be inactivated by various photosensitizers, including methylene blue [7], merocyanine 540 [8], hypericin and Rose bengal [9], porphyrins [10], and phthalocyanines [11,12]. Although all viral components can serve as potential molecular targets for ROS, the most readily available are proteins and unsaturated lipids of viral envelopes [13]. As summarized by Costa et al. [14], the main types of photodynamic damage to mammalian virus envelopes include: altering protein cross-link formation, a complete loss of proteins, changes in protein conformation or the alteration of molecular mass and charge. These forms of virion damage can prevent viral absorption and host penetration, inhibit membrane fusion, and reduce infectivity. The kinetics of viral inactivation depends on the photosensitizer concentration, yield of singlet oxygen, and parameters of irradiation [9,14].

Phthalocyanines with intensive absorption at the far red to near-infrared wavelengths are commonly used photosensitizers. Energy migration from the triplet excited state of phthalocyanine to the triplet ground state of molecular oxygen leads to the formation of highly reactive singlet oxygen [15]. Peripherally charged substituents prevent aggregation, greatly improve compatibility of metallophthalocyanines (Me-Pcs) with aqueous media and increase singlet oxygen yield generation up to 0.60–0.65 values for octa-substituted compounds [16]. Cationic substituted Me-Pcs with long triplet lifetimes, high photostability, and high yields of singlet oxygen are potent antimicrobial photosensitizers against both gram-positive and gram-negative bacteria, pathogenic fungi, and viruses [17,18,19].

As previously demonstrated in vitro, octacationic octakis(cholinyl) zinc phthalocyanine (Zn-PcChol8+) is highly efficient in disinfecting water from avian influenza A virus, H5N1 subtype [20]. In this instance, photodynamic treatment with Zn-PcChol8+ (1.0 mg/L) and white light (15 J/cm^2^) completely inactivated H5N1 in all virus titers tested by up to 7.0 lg EID50, which is more effective compared to methylene blue or proflavine acetate. The singlet oxygen induced inactivation of viral fusion suggested hemagglutinin [9], the viral fusion surface glycoprotein, to be one possible target for photodynamic treatment. Zarubaev et al. [21] showed fragmentation of membranes and loss of surface glycoproteins in H1N1 influenza virus inactivated in allantoic fluid with 0.5 mg/mL fullerene photosensitizer and 5 h white light irradiation. However, it remains unclear whether inactivation is due to the loss of glycoproteins or due to oxidative destruction of the virus membrane. Also, long irradiation times in allantoic fluid can cause other side effects yet to be identified. To determine what ultrastructural components of influenza virions are target by PDI, we inactivated highly pathogenic avian H5N8 influenza virus with the photosensitizer Zn-PcChol8+ at different concentrations (2 or 4 µM that corresponds to 3.2 or 6.4 µg/mL) with 20 min white light irradiation and studied the associated morphological changes using a transmission election microscope (TEM).

## 2. Materials and Methods 

### 2.1. Virus Production and Purification 

Avian influenza A virus A/domestic duck/Siberia/103/2016 (H5N8) was grown in 10-day old embryonated chicken eggs. After incubation at 37°C for 3 days, the allantoic fluid was harvested and stored at −80°C until use [22]. All H5N8 experiments were performed according to the standard protocols at the BSL 3 Laboratory (Federal Research Centre fundamental and translational medicine, Novosibirsk, Russia).

Initially, the protocol did not contain a purification step but attempted to observe the effects of PDI using non-purified allantoic fluid. However, the allantoic fluid appears to mask viral particles because of the high concentration of proteins and other biomolecules. Therefore, we used a simple and rapid variation of the purification method developed by [23] for bacteriophages. In brief, the allantoic fluid was filtered through a 0.22 μm filter and diluted five times with phosphate-buffered saline (PBS, pH 7.4). Centrifugal concentrators “Vivaspin 6” MWCO 300 kDa (Vivaproducts, Littleton, MA, USA) were filled with the suspension (6 mL per tube) and centrifuged at 4000× *g* for 5 min. PBS was then added up to 6 mL and the concentrators were vigorously mixed and centrifuged again at the same speed and duration as above. This procedure was repeated five times. After the fifth centrifugation step, the suspension was collected from the concentrators (about 1 mL per tube). This method provides high quality purification but leads to a reduction in virus titer (from 8.375 ± 0.42 to 7.125 ± 0.34 in our preparation) because some virions can get stuck in the membrane [24].

### 2.2. Cell Culture and Virus Titration

Madin Darby Canine Kidney (MDCK) cells were grown at 37 °C with 5% CO_2_ in Eagle minimal essential medium (Thermo Fisher Scientific, Waltham, MA, USA, supplemented with 10% fetal bovine serum (FBS, Invitrogen) and gentamicin 50 μg/mL (Biolot, St. Petersburg, Russia). After virus inoculation, cells were cultured in Eagle minimal essential medium supplemented with 0.2% bovine serum albumin (BSA, Biolot), gentamicin 50 μg/mL (Biolot) and 2 μg/mL of trypsin (Sigma-Aldrich, St. Louis, MO, USA). In all experiments, cells that were not infected with the virus were included as controls.

For virus titration, MDCK cells were grown to 90% confluency in 96-well plates, then washed with PBS and infected with 10-fold serial dilutions (from 10-1 to 10-8) of the treated or non-treated virus. After incubating for 30 min at 37 °C, the supernatant was removed and cells were incubated with 200 μL of virus growth medium at 37 °C, 5% CO_2_ for 5 days. Virus-induced cytopathic effect was detected and virus titers were determined as the 50% tissue culture infectious dose (TCID50) per ml according to Reed and Muench [25]. To prevent accidental irradiation of samples containing photosensitizer, the plates were protected from light with aluminum foil.

Singlet oxygen is a nonradical reactive oxygen species that readily oxidizes aromatic amino acids, unsaturated lipids and other biomolecules, and has the extremely short lifetime (about 1 μs) in biological systems [26]. It seems unlikely that traces of singlet oxygen could be detected after photodynamic treatment which could negatively impact MDCK cells. Moreover, MDCK cells were infected with 10-fold dilutions (from 10^−1^ to 10^−8^) of treated virus which further minimizes the effect of residual products of photodynamic reaction.

### 2.3. Photodynamic Inactivation

Zn-PcChol8+ synthesized in the State Scientific Center “NIOPIK” (Russia) was used as a photosensitizing agent for virus inactivation in two treatments corresponding to concentrations of either 2 or 4 µM. The irradiation system was constructed from 30 W halogen lamp with collimated light beam (12 mm diameter). Virus suspensions (1 mL) were irradiated in opened 1.5 mL “Eppendorf” vials for 20 min at light dose of 12 J/cm^2^. The intensity of light reaching the probes was measured with PM160T Wireless Power Meter (THORLABS GmbH, Dachau, Germany) and made up 10 mW/cm^2^. The vial containing the virus suspension was then placed in a plastic water filled box to prevent overheating. In addition, we included two control treatments: dark (incubated in the dark with photosensitizer) and light (irradiated as above without photosensitizer). 

### 2.4. Electron Microscopy

A 200 mesh copper TEM grid (SPI Supplies, West Chester, PA, USA) with carbon stabilized ultrathin (invisible on the water surface) [27] formvar support film was placed on a 10 µl droplet of suspension for 30 s and then dried using filter paper. A 10 µl droplet of uranyl acetate water solution (1% *w*/*v*) was then added to the grid surface for 15 s. After drying, using filter paper again, the grid was examined with a TEM (JEM-1400; Jeol, Japan) at an accelerating voltage of 80 kV. 

Three TEM grids per sample were prepared independently to avoid random artefacts. At least 100 virions were examined in each sample. To prevent accidental irradiation, the preparation of the dark control was conducted in a dark room.

## 3. Results

### 3.1. Purified and Non-Purified Allantoic Fluid Comparison

**Figure 1:**
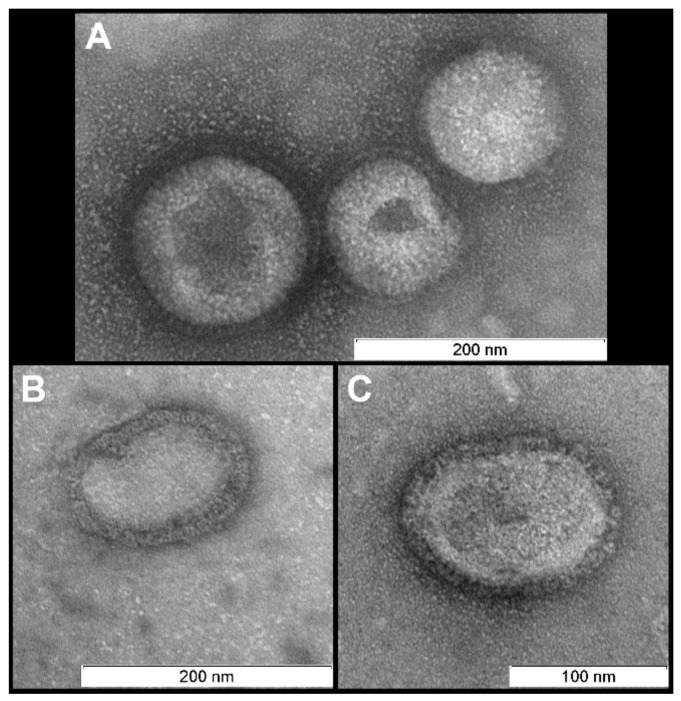
Virions after purification. Morphology is typical for influenza virus: near-spherical enveloped virions (diameter 100–130 nm) with spikes. Scale bar 200 nm (**A**,**B**) and 100 nm (**C**).

Using a non-purified allantoic liquid with 2 µM of photosensitizer proved to be inadequate because it was difficult to observe any structural features. This is likely due to the high concentration of proteins and other biomolecules within allantoic fluid which mask the virions. In addition, these molecules react with the singlet oxygen produced by the photosensitizer. TEM images revealed that purification of the allantoic fluid dramatically improved the visibility of virion structures (Figure 1). The concentrator-based virus purification technique is faster and easier compared with classical ultracentrifugation-based protocols and produced pure high-quality viral samples with intact virions. Thus, non-purified allantoic fluid cannot be used for ultrastructural examination of influenza virus after photodynamic inactivation and it is necessary to purify the virus suspension for accurate visualization.

### 3.2. H5N8 Photodynamic Inactivation and Infectivity

**Figure 2:**
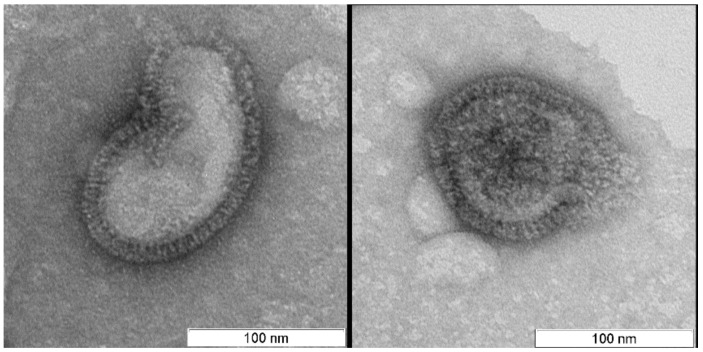
Light control. Virions look intact. Scale bar 100 nm.

**Figure 3:**
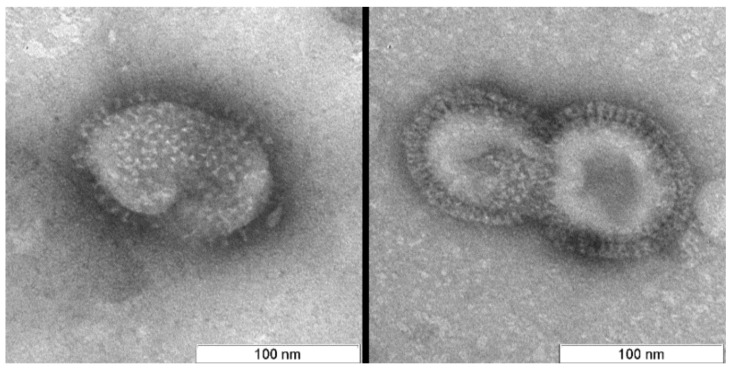
Dark control. Virions look intact. Scale bar 100 nm.

**Figure 4:**
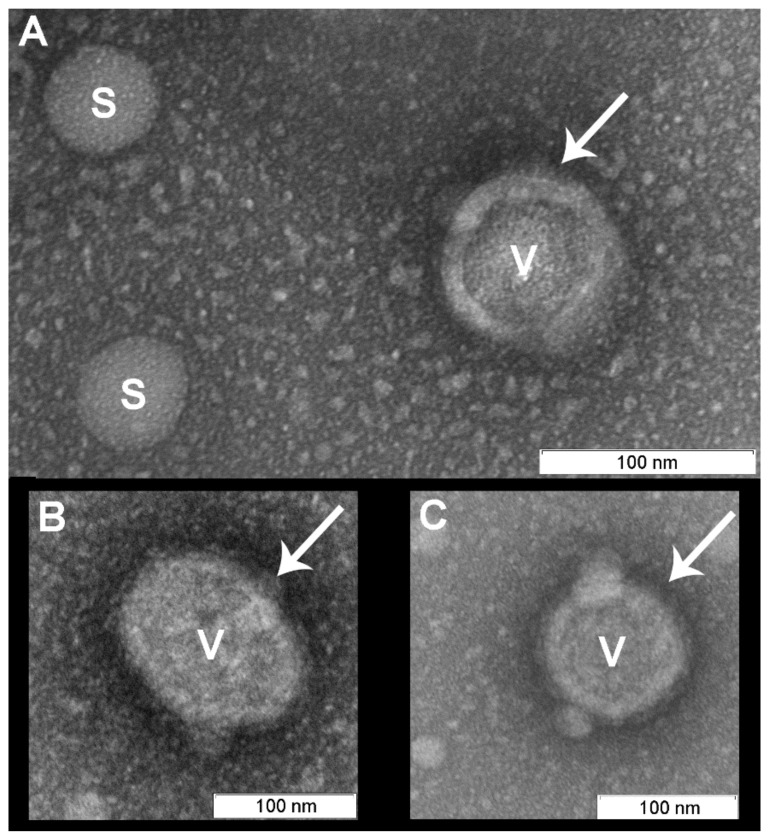
Virions (“V”) irradiated for 20 min with photosensitizer (2 µM) remained structurally normal with membranes maintaining their structural integrity. The spikes appeared to be absent from the viral membranes, however the “halo” may indicate the presence of glycoproteins (marked with arrows). Spherical vesicles (diameter 30–50 nm, marked “S” (**A**), the similar structures can be recognized on virions surface (B, C)) and grain-like small (5–15 nm) structures were visible and were not observed in the controls. Scale bar 100 nm.

**Figure 5:**
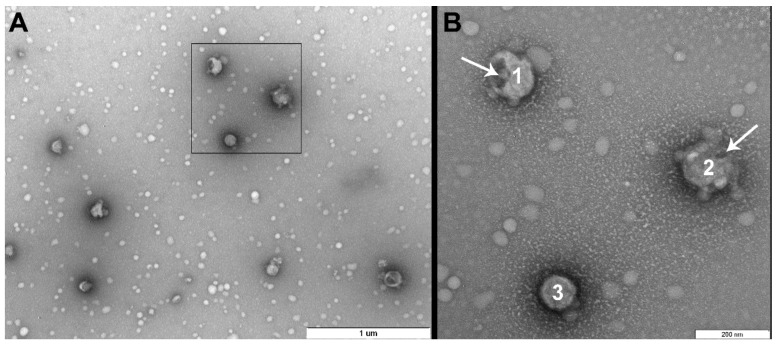
Virions after photodynamic inactivation (PDI) (4 µM photosensitizer). (**A**) Low magnification (scale bar 1 µm); (**B**) Magnified area denoted by black box in (A) (scale bar 200 nm). It is possible to distinguish two different forms of “bald” virus particles: smooth spherical virions (“3”) and virions with damaged membranes (“1” and “2”). Membrane damage or “dark windows” are marked with arrows.

**Figure 6:**
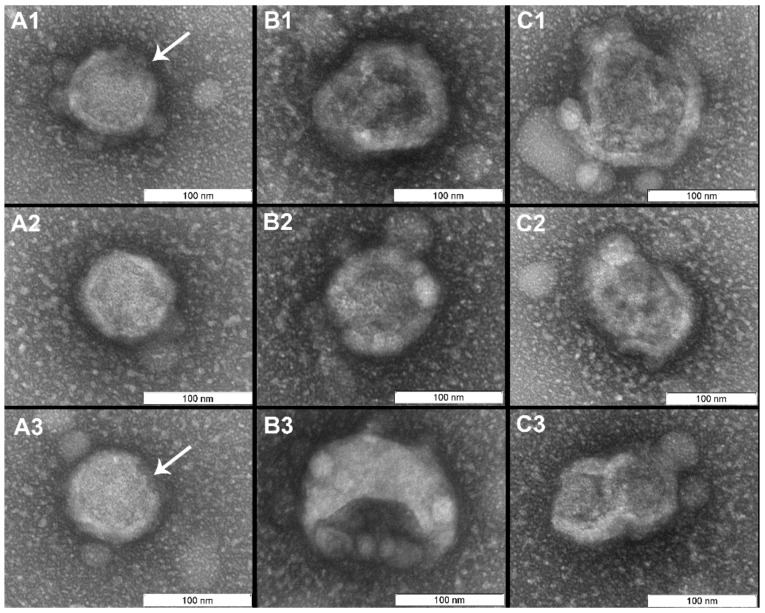
Virions after PDI (4 µM photosensitizer). All virus particles are “bald”. Columns are organized according to the condition of the lipid membranes: (**A**) membranes maintained structural integrity, minor membrane damages marked with arrows; (**B**) virions with partially destroyed membranes; (**C**) membranes are destroyed and virions disintegrated. Scale bar 100 nm.

Five samples of H5N8 influenza virus were examined with a TEM under different conditions:

- purified suspension without any treatment (Figure 1);
- irradiated without photosensitizer (light control, Figure 2);
- not irradiated (dark control) suspension with 4 µM of the photosensitizer (Figure 3);
- irradiated with photosensitizer at concentrations of 2 µM (Figure 4);
- irradiated with photosensitizer at concentrations of 4 µM (Figure 5 and Figure 6).

There were no differences in viral morphology between untreated virions or light and dark controls (Figure 1, Figure 2 and Figure 3).

**Figure 7:**
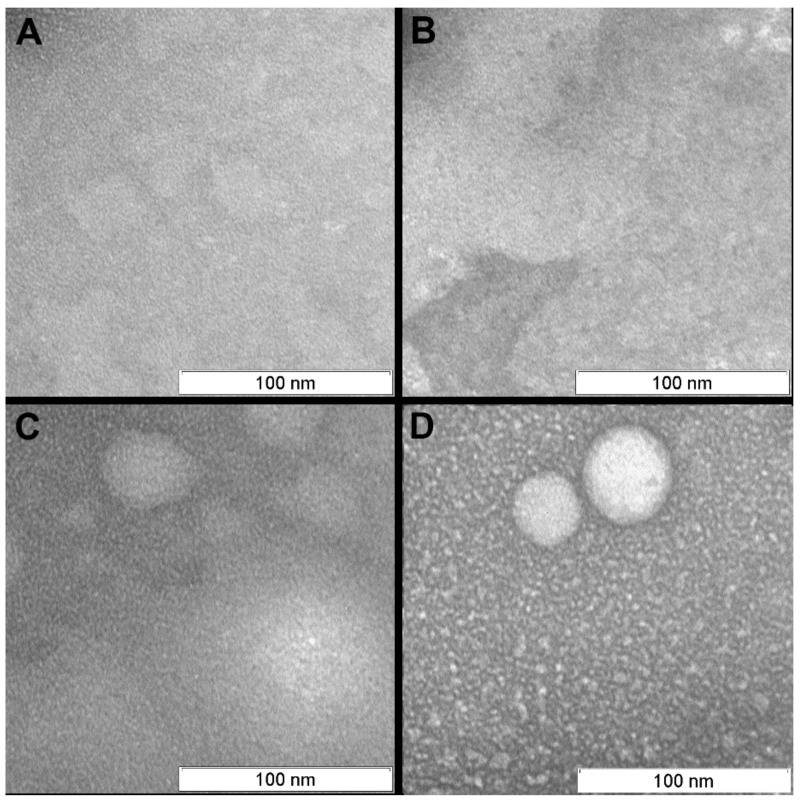
Background of the transmission electron microscopy (TEM) pictures (area between virions). (**A**) Purified virus suspension; (**B**) Light control; (**C**) Dark control; (**D**) After photodynamic inactivated (4 µM photosensitizer, 20 min). Scale bar 100 nm.

In contrast to the controls, virions irradiated with photosensitizer were damaged (Figure 4, Figure 5 and Figure 6). Virions were completely inactivated (Table 1) for both photosensitizer concentrations (2 and 4 µM) and all lost their surface glycoproteins (spikes). Virions inactivated with 2 µM of the photosensitizer became “bald”, but their membranes appeared to maintain structural integrity and were coated with a “halo”, which can point to non-complete oxidative destruction of the glycoproteins (Figure 4). Evidence for the maintenance of membrane structural integrity is given in Figure 4 which shows virions keeping their normal near-spherical shape and remaining impermeable to the negative stain [28]. We also observed smooth spherical vesicles and small grain-like structures when using photosensitizer (2 and 4 µM concentrations: Figure 4, Figure 5 and Figure 6) which were not present in the controls (Figure 7).

The virus suspension inactivated with 4 µM of photosensitizer contained three forms of “bald” virions: smooth spherical virus particles (~25% of examined virions), virions with partially destroyed membranes (“dark windows”, ~60%), and completely (deformed, disintegrated particles, ~15%) destroyed membranes (Figure 5 and Figure 6). These forms seem to reflect the degree of virion damage and may be associated with both irradiation fluctuations and the heterogeneity of virions with respect to photosensitizer binding. Long periods of irradiation with high concentrations of photosensitizer destroyed all virions as shown in other studies [21].

Virus titer was determined by a virus titration using MDCK cells. The virus titration showed that the titers of non-treated purified virions and controls were similar (Table 1). The titer decreasing by purification can be explained by the loss of the virions in the centrifugal concentrator pores. In samples irradiated with photosensitizer (both concentrations), complete inactivation was achieved (Table 1). This suggests that even the “bald” virions with membrane integrity maintained are non-infectious and that a complete loss of virion integrity is not necessary for virus inactivation. This finding implies that PDI can be used for manufacturing “bald” virions which are non-infectious but likely maintain structural membrane integrity and RNA. 

On the micrographs of both (2 and 4 µM of photosensitizer) photodynamically treated samples smooth spherical vesicles (30–50 nm) and small (5–15 nm) grain-like structures have been detected (Figure 4). Concentration of both types of structures is higher in the sample irradiated with 4 µM of photosensitizer. Such structures were not presented in controls (Figure 7). 

## 4. Discussion

**Figure 8:**
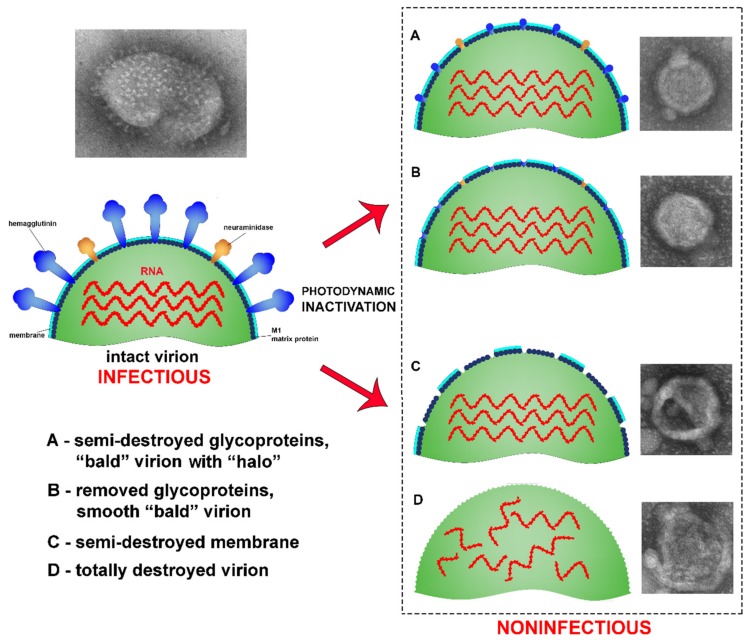
An illustration of the effects photodynamic inactivation has on influenza virus. (**A**) semi-destroyed glycoproteins;(**B**) removed glycoproteins; (**C**) Partial membrane destruction; (**D**) Total virion destruction.

Here, we investigated the ultrastructural properties of photodynamic inactivation of H5N8 influenza virus. We demonstrate that PDI treatment of the virus induces the removal of viral surface glycoproteins and makes virions non-infectious even without membrane damage. Because proteins are readily oxidized by singlet oxygen [13], hemagglutinin, the viral fusion surface glycoprotein, might be particularly susceptible to the singlet oxygen produced by PDI. Overall, three distinct forms of virion damage were induced by photodynamic inactivation. We observed “bald” virions with membranes keeping structural integrity, virions with damaged membranes and virions that were totally destroyed (Figure 8). We show that all three forms are non-infectious (Table 1). 

In this study, the treatment with the photosensitizer Zn-PcChol8+ (2 or 4 µM) and 20 min white light irradiation (light dose 12 J/cm^2^) rendered H5N8 virions unable to infect MDCK cells. Such a strong inactivation effect with this octacationic phthalocyanine photosensitizer is not unique to the influenza virus H5N8 as it has been previously shown for the H5N1 subtype [20]. Another tricationic phthalocyanine inactivated H1N1 with IC50 value 0.087 nM [18] after 20 min red light illumination (light dose 48 J/cm^2^). Thus, cationic phthalocyanines are highly active photosensitizers towards influenza viruses.

“Bald” influenza virions were first described by Brand and Skehel in 1972 [29] and were created using a protease treatment (bromelain). Bromelain enzymatically detaches ectodomains of hemagglutinins which in the untreated viruses form spikes protruding from the membrane surface. On negatively stained electron micrographs, bromelain treated virions generally appear smooth with no visible spikes [30]. A similar pattern was observed in this study (Figure 4, Figure 5 and Figure 6), whereby irradiated H5N8 virions treated with Zn-PcChol8+ appeared “bald”. This suggests that ROSs can detach spike glycoproteins. Such “bald” virus particles can be used in studies of biophysical properties of virions (e.g., remove surface glycoproteins to estimate their influence on virion stiffness [30]) and in viral engineering. 

The smooth vesicles and small grain-like structures detected in the photodynamically treated samples can be formed from detached virus subunits. Such structures were not presented in control samples. The vesicles looked similar to structures found on the H3N2 viral particles exposed in acid medium [31]. The “bald” virions were larger and had a stained dark core, thus they appear distinct from the smooth round vesicles on the micrographs. 

Using a high photosensitizer concentration (4 µM), we observed irregular virion shapes which likely reflect virion membrane damage. One explanation is that the hydroperoxides of cholesterol and other lipids disrupt viral membranes [32] and induce lipid membrane damage. 

In conclusion, influenza virus in our experiments was inactivated with singlet oxygen that was generated by the photoexcited phthalocyanine photosensitizer. Furthermore, our results demonstrate that the detachment of the influenza virus surface glycoproteins and the loss of infectivity by “bald” virions was the result of the photodynamic treatment. This type of viral inactivation may be representative for other sources of singlet oxygen. Indeed, the highly reactive ROS produced by chemical reactions in the dark [33] and even by immune cells during oxidative burst [34] also exhibits viricidal properties.

## Figures and Tables

**Table 1:** Determination of virus titers by virus titration on Madin Darby Canine Kidney (MDCK) cells. It has been shown that 20 min irradiation with photosensitizer (PS) leads to the total inactivation of the virus.

Titer of Virus	Allantoic Fluid	Purified Suspension	Stored in Dark with PS (4 µM)	Irradiated for 20 Min without PS	Irradiated for 20 Min with PS (2 µM)	Irradiated for 20 Min with PS (4 µM)
lgTCID50/mL ± 2Ϭ	8.375 ± 0.42	7.125 ± 0.34	7.25 ± 0.30	7.0 ± 0.42	0	0
